# Accuracy of BMI correction using multiple reports in children

**DOI:** 10.1186/s40608-016-0117-1

**Published:** 2016-09-13

**Authors:** Madhumita (Bonnie) Ghosh-Dastidar, Ann C Haas, Nancy Nicosia, Ashlesha Datar

**Affiliations:** 1 RAND Corporation, Department of Economics and Statistics, 1776 Main Street, Santa Monica, CA 90401 USA; 2 RAND Corporation, Department of Economics and Statistics, 4570 Fifth Ave, Pittsburgh, PA 15213 USA; 3 RAND Corporation, Department of Economics and Statistics, Boston, MA USA; 4 Dornsife Center for Economic and Social Research (CESR), University of Southern California, Los Angeles, CA USA

**Keywords:** BMI, Linear correction, Height, Weight, Measurement error, Obesity, Children

## Abstract

**Background:**

Errors in reported height and weight raise concerns about body mass index (BMI) and obesity estimates obtained from self or proxy reports. Researchers have corrected BMI using linear statistical models, primarily with adult samples. We compared the accuracy of BMI correction in children for models that included child or parent reports versus both reports, and models that separately predicted height and weight compared to a single model for BMI.

**Methods:**

Height and weight from child reports, parent reports, and objective measurements for 475 children participating in the Military Teenagers’ Environment, Exercise and Nutrition Study were analyzed. Two approaches were evaluated: (1) separate linear correction models for height and weight versus (2) a single linear correction model for BMI. Each approach considered models for height, weight, or BMI with child reports, parent reports, or both reports, respectively, as predictors, stratified by gender. Prediction accuracy was computed using leave-one-out validation. Models were compared using root mean squared error for BMI, and sensitivity and specificity for overweight and obesity indicators.

**Results:**

Models that included both reports provided the best fit relative to a model using either set of reports, with adjusted R^2^ of height, weight, and BMI models ranging from 67.1 to 87.6 % in males, and 69.2 to 88.3 % in females. Estimates of BMI from separate models for height and weight had the least prediction error, relative to those derived from a single model for BMI or from uncorrected (child or parent) reports. Cross-validated Root Mean Squared Error (RMSEs) preferred a model that included only parent reports among males and females, compared to models with only child reports or both reports. When assessing sensitivity (true positive) for obesity and overweight/obesity, the results varied by gender and outcomes. Specificity (true negative) was similarly high for all models.

**Conclusion:**

Objective measurements are more accurate than self- or proxy-reports of BMI. In situations where objective measurement is infeasible, an approach that combines collecting a validation sub-sample including multiple reports of children’s height and weight, with estimation of BMI correction models maybe a cost-effective and practical solution. Correction models generate BMI estimates that are closer to objective measurements than reports.

## Background

Obesity is “one of the most serious public health challenges of the 21st century” [1, 2]. It has more than doubled among children during the past 30 years [3, 4]. High rates of obesity in children are particularly concerning because of the potential for long-term health consequences, such as increased risk of heart disease, type 2 diabetes, high blood pressure and other chronic conditions [5]. Thus, ongoing monitoring of overweight and obesity is important. Body mass index (BMI, weight [kg]/height [m^2^]) has become the most common indicator to assess obesity because it is an inexpensive and noninvasive surrogate measure of body heaviness, shown to exhibit high correlations with body fat and future health risks [6–9].

The three standard ways to collect height and weight data are: 1) self-report, 2) proxy-report (often by a parent), and 3) in-person objective measurement. While objective measurements conducted by trained personnel are superior to self- or proxy-reports, they are expensive to collect, especially in large or dispersed samples [9]. In an environment of budget cuts and declining response rates [10], many national surveys including the Behavioral Risk Factor Surveillance System [11], the National Health Interview Survey [12], and the National Survey of Children’s Health [13] primarily rely on parental reports of children’s height and weight. Yet reported height and weight are shown to include non-trivial amounts of measurement error. For example, BMI was 0.7 kg/m^2^ higher when based on parent-reported values vs measured height and weight [14]. A review of the literature found that sensitivity of reported BMI for screening for overweight ranged from 55 to 76 %, and that overweight prevalence was −0.4 to −17.7 % lower when BMI was based on self-reported versus directly measured data for adolescents [15].

Concern about measurement error has fostered an emerging literature on how less costly data collection efforts can be utilized in BMI studies. One cost effective approach is the use of BMI “correction models” [16–22]. A “correction model” can be estimated with a dataset that includes both reports and measurements. The estimated model coefficients can then be applied in other studies which have collected reported data only to produce corrected BMI estimates. Past efforts have largely focused on self-reports in adult samples. The quality of BMI correction models has not been fully explored with other sub-populations (e.g., children), where the nature of reporting errors might differ and where proxy reports are more common. Further, most studies have ignored the issue of out-of-sample prediction and *validity shrinkage* [23], and do not provide a good understanding of predictive performance of such models.

The response error mechanism underlying misreporting of height and weight can be random or systematic. While some respondents may not know their actual height or weight, others may intentionally misreport to fit social norms. An additional complication specific to BMI is that the error mechanism may differ for height and weight. A review paper summarizing multiple studies found that self-reported height in adolescents was both underestimated and overestimated with reporting biases ranging from −1.1 to 2.4 cm, while reported weight was usually underestimated [15]. Results from earlier studies also suggest that girls tend to underestimate their weight more so than boys [15]. Thus, separate correction models for height and weight stratified by gender [24] may be superior to a single model for BMI.

An additional issue with surveys of children is that they typically ask for parental reports of height and weight, in lieu of children’s self-reports. The magnitude of response biases has been found to differ for self- versus proxy-reports in adults [25]. One recent study in Quebec actually collected child- and parent-reports for children between 8 and 12 years of age, and found that the two were comparable. Both child and parent reports underestimated the children’s weight by 1 kg, height by less than 1 cm, and BMI by less than 0.25 kg/m^2^, on average [26]. Given the lack of multiple studies comparing child and parent reports, it is not well understood whether collecting two sets of reports of height and weight has added value.

The Military Teenagers’ Environment, Exercise and Nutrition Study (M-TEENS) provides a unique opportunity to explore these issues. A subsample of M-TEENS respondents completed objective measurements, in addition to providing child- and parent-reports of child’s height and weight. This is the only national sample in the U.S. that we know of that includes child and parent reports of height and weight, in addition to measurements, for youth (12–13 year olds). As a result, it provides a unique chance to conduct a *validation study*. The aim of this study is to propose a simple, linear BMI correction model which does not require many additional predictors, and can be estimated with small validation samples. We also tested two hypotheses – (i) a model that predicts BMI using both parent and child reports will be more accurate than a model that includes only one set of reports; and (ii) separate correction of height and weight to derive BMI will improve prediction accuracy compared to a model that directly predicts BMI because the reporting errors for height and weight may differ. We explored both hypotheses using measures of predictive performance.

## Methods

### Design and sample

M-TEENS was designed to assess how the food and physical activity environment in schools and neighborhoods may influence the diet, physical activity, and BMI of children ages 12–13. The study surveyed children and their parents/guardians (hereafter, parents) from families of enlisted Army personnel located at 12 Army installations across the four U.S. Census regions. Using the Army’s records, M-TEENS staff contacted families who were located at these installations for at least 18 months and who likely had a 12–13-year-old (as of March 31, 2013) in the household. The Defense Manpower Data Center (DMDC) provided participants’ contact information. We attempted to contact a larger than necessary sample for several reasons. First, military families move frequently due to periodic re-assignment, so that their contact information is not always updated in a timely manner. Second, information on members’ active duty status does not reflect recent separations from the military. Lastly, response rates in military samples are considerably lower than in civilian samples [27, 28].

Of 8,545 enlisted personnel that were initially emailed and/or mailed recruitment letters, 2,106 completed an eligibility screener. Families were eligible to participate if they met three conditions. First, the service member did not intend to leave the military within the coming year. Second, the 12- or 13-year-old resided with the enlisted parent at least half-time. Finally, the 12- or 13-year old child was enrolled in a public or Department of Defense Education Activity school. Of those screened, 1,794 (85 %) households were eligible and 1,188 (66 %) provided consent.

Between Spring 2013 and Winter 2013/2014, 1,022 surveys were completed online. During data collection, study personnel visited each installation for three days to collect objective measurements for those with a survey completed, with families participating on a walk-in basis. Of the 1,022 survey respondents with at least a parent or child survey response, 521 completed an objective measurement. The sample size of children with self-reports, parent-reports, and measurements of height and weight is 475. The limited window for objective measurements lowered the number of measurements, while living on-installation increased the chances of completing the measurements. This study was approved by the Institutional Review Boards at RAND, University of Southern California, and the Army Human Research Protection Office. Online consent and assent was obtained from parents and children, respectively. All sampled, enlisted persons were sent instructions to their Department of Defense email address with unique login credential and a unique link to the consent form generated specifically for the sampled person.

### Measures

#### Objective measurements

Height and weight were measured by trained personnel at each installation. Height was measured using a Seca 213 stadiometer, rounded to the nearest 0.1 cm. Weight was measured using a Tanita UM-041 F digital scale, recorded to the nearest 0.1 kg. All measurements were taken at least twice; a third measurement was taken if the two measurements differed by a pre-determined amount (>0.5 cm for height, >0.2 kg for weight). The average of the two closest measurements was used. About 90 % of measurements were conducted within one month of the survey.

#### Reported height and weight

The child (parent) survey included the following questions: “how tall are you (is your child) without shoes on?” and “how much do you (does your child) weigh without shoes on?” The reporting units are inches and feet for height and pounds for weight. Child- and parent-reported BMI were computed as the ratio of child- and parent-reported weight (kg) and height-squared (m^2^), respectively.

#### BMI, obesity

BMI was computed as the ratio of measured or reported weight [kg] to height[m]-squared. The child’s age and gender were used to calculate BMI percentile using the 2000 Centers for Disease Control BMI-for-age growth charts [29]. We constructed indicators of obesity and overweight/obesity, where obesity is defined as BMI in the 95^th^ percentile or higher and overweight is defined as BMI between the 85 and 95^th^ percentiles.

#### Participant characteristics

The parent survey asked about the child’s race/ethnicity. Gender was obtained from child survey; birthdate came from DMDC records. Age in months is the difference between the measurement date and the birthdate and rounded to the nearest month.

### Analysis

We summarized the distributions of reported and measured outcomes for the overall sample, and by gender for the analysis sample (*n =* 475). To identify biases in reporting, we tested for significant differences and correlations between objective and reported values of height, weight and BMI using a paired *t*-Test and Fisher’s *Z*-Test, respectively. Similarly, we tested for agreement between reports and measurements of binary variables such as obesity or overweight/obesity using McNemar’s test.

A linear regression model with measured height, weight, or BMI as the dependent variable and reported values of the same variable as the key independent variable(s) was used for correction. Three models were considered per outcome – Model 1 included parent report only, Model 2 included child report only, and Model 3 included both child and parent reports, estimated separately for boys and girls. The reported variables were centered before they were entered as linear and quadratic terms. Age (in months) and race-ethnicity indicators were included in all models [21]. The quality of models was assessed using adjusted-*R*^2^ and Akaike Information Criterion (AIC) [30]. The AIC is proportional to the log likelihood penalized by the number of model parameters, and used as a measure of the relative quality of statistical models.

Typically, regression coefficients from a BMI correction model estimated with a validation sample are applied to another dataset which only includes reported height and weight. As Ivanescu et al. [23] have underscored, the true test of a model’s predictive capacity can be assessed when the model is tested on an independent dataset that was not used to develop the model; cross-validation approaches provide an objective assessment of out-of-sample prediction accuracy, accounting for both the bias and variance of model predictions. Thus, we provide estimates of prediction error that are obtained from leave-one-out cross validation [31]. In particular, leave-one-out cross validation is well suited to smaller sample sizes (e.g. M-TEENS) [23].

Corrected BMI was computed from predicted height and weight (‘*indirect*’ approach), or directly predicted using reported BMI (‘*direct*’ approach). We computed RMSE associated with the continuous outcome (BMI). We also assessed classification error using *sensitivity* and *specificity* for the binary indicators of obesity and overweight/obesity. *Sensitivity* (true positive) indicates a model’s ability to correctly classify an individual as overweight or obese while *specificity* (true negative) indicates its ability to correctly classify an individual as non-obese or non-overweight/non-obese [32]. Further, we compared the corrected estimates to uncorrected reports to understand whether correction improves upon use of self- or parent-reports. To account for sampling error, RMSE values within 0.05 units of each other and sensitivity and specificity estimates within 3 % of each other are treated as similar and not discussed. All analyses were performed in SAS software, Version 9.4 of the SAS System for Windows.

## Results

### Sample characteristics

In the analysis sample of 475 with validation data, 46 % were female, 43 % non-Hispanic white, 21 % non-Hispanic black, and 24 % Hispanic/Latino (Table 1, column 1). The average age was 157 months (13.1 years). The average measured height and, weight were 159 cm and 53 kg, respectively. The average BMI was 20.8 kg/m^2^. The percent obese and percent overweight or obese, derived from measured BMI, were 10.7 and 28.0 %, respectively.

**Table 1:** Characteristics of M-TEENS validation sample

	Total, *N* = 475	Boys, *N* = 258	Girls, *N* = 217
Age (months), mean (SD)	156.8 (7.3)	157.2 (7.4)	156.2 (7.0)
Female, %	45.7	n/a	n/a
Race/Ethnicity			
White non-Hispanic, %	42.5	42.3	42.9
Black non-Hispanic, %	21.3	22.1	20.3
Hispanic or Latino, %	24.0	24.8	23.0
Other^a^, %	12.2	10.9	13.8
Objective measures			
Measured height (cm), mean (SD)	159.2 (8.4)	160.2 (9.3)	158.0 (6.9)
Measured weight (kg), mean (SD)	53.1 (12.6)	52.8 (12.8)	53.6 (12.2)
Measured BMI, mean (SD)	20.8 (4.0)	20.4 (3.9)	21.3 (4.1)
Obese, %	10.7	12.0	9.2
Overweight or obese, %	28.0	25.2	31.3
Parent report			
Parent-reported height (cm), mean (SD)	157.8 (10.1)^b^	158.6 (11.1)^b^	156.8 (8.6)^b^
Parent-reported weight (kg), mean (SD)	50.8 (12.2)^b^	50.7 (12.4)^b^	50.9 (12.1)^b^
Parent-reported BMI, mean (SD)	20.3 (4.0)^b^	20.0 (3.8)^c^	20.6 (4.2)^b^
Obese, %	9.5	9.7	9.2
Overweight or obese, %	25.5	27.1	24.1^d^
Child report			
Child-reported height (cm), mean (SD)	158.3 (10.2)^b^	158.8 (11.7)^c^	157.8 (8.1)
Child-reported weight (kg), mean (SD)	51.2 (12.4)^b^	51.1 (12.7)^b^	51.3 (12.1)^b^
Child-reported BMI, mean (SD)	20.3 (4.0)^b^	20.1 (3.8)	20.5 (4.1)^b^
Obese, %	8.6^e^	9.3^e^	7.8
Overweight or obese, %	23.8^d^	25.2	22.1^d^

*M-TEENS* Military Teenagers’ Environment, Exercise and Nutrition Study, *BMI* body mass index, *SD* standard deviation; ^a^ includes multiracial, Asian, American Indian/Alaska Native, Native Hawaiian/Pacific Islander; ^b^ One-sample *t*-Test *P* <.001; ^c^ One-sample *t*-Test *P* <.01, testing parent and child reported height, weight and BMI against objective measurement; ^d^ McNemar’s test *P* <.01; ^e^ McNemar’s test *P* <.05 comparing parent and child reported obesity or overweight/obesity against objective measurements

### Reporting biases

On average, child-reported and parent-reported heights were 0.9 cm lower (*P* <.001*)* and 1.4 cm lower (*P* <.001), respectively, than measured height (Table 1). Child-reported and parent-reported weights were 1.9 kg lower (*P* <.001*)* and 2.3 kg lower (*P* <.001*)* than measured weight. Also, child-reported and parent-reported BMI were both 0.5 units lower than measured BMI (*P* <.001). The estimates of obesity and overweight/obesity derived from child reports were 2.1 and 4.2 percentage points (hereafter, pp) lower, respectively, than the measured values. The child-reported estimates of the binary indicators were less accurate, on average, compared to parent-reports (which were 1.2 and 2.5 pp lower, respectively). Additional information about the distribution of bias is provided in the Appendix.

Among males, child- and parent-reports of height were 1.4 cm (*P* <0.01) and 1.6 cm (*P* <0.001) lower than the measured value, respectively. Child- and parent-reported weights were 1.7 kg lower (*P* <.001) and 2.1 kg lower than measured weight (*P* <.001). Also, child-reported BMI was 0.3 kg/m^2^ lower while parent-reported BMI was 0.4 kg/m^2^ lower than measured BMI (*P* <.01). Obesity was underestimated by 2.7 pp using child-reports. Obesity was underestimated by 2.3 pp, while overweight/obesity was over-estimated by 1.9 pp using parent-reports. Among females, parent-reported height was 1.2 cm lower than measured height (*P* <.001). Child-reported and parent-reported weight were 2.3 kg lower (*P* <.001) and 2.7 kg lower (*P* <.001), respectively, than measured weight. Also, child-reported BMI was 0.8 kg/m^2^ lower (*P* <.001) and parent-reported BMI was 0.7 kg/m^2^ lower (*P* <.001) than measured BMI. Obesity and overweight/obesity were underestimated by 1.4 and 9.2 pp, respectively, using child reports. While overweight/obesity was underestimated by 7.2 pp using parent reports, obesity alone was not underestimated. The magnitude of bias was greater when using self-reports than parent-reports among females.

### Association of reported and measured outcomes

In Table 2, we explored the correlations among parent reports, child reports, and measurements of weight, height, and BMI. The correlations between reported and measured weight were higher (*r =* 0.93) than that between reported and measured height (*r* = 0.79–0.82) and BMI (*r =* 0.87). The patterns of association for males and females were similar, with slightly higher correlation between measured and reported height and BMI among females. All correlations were statistically significant (*P* <0.001).

**Table 2:** Correlation of reported and measured height, weight and BMI in M-TEENS

	Measured vs. Reported Weight	Measured vs. Reported Height	Measured vs. Reported BMI
Parent report	0.93^a^	0.79^a^	0.87^a^
Child report	0.93^a^	0.82^a^	0.87^a^
Males			
Parent report	0.92^a^	0.78^a^	0.85^a^
Child report	0.93^a^	0.82^a^	0.86^a^
Females			
Parent report	0.93^a^	0.82^a^	0.89^a^
Child report	0.92^a^	0.85^a^	0.88^a^

*M-TEENS* Military Teenagers’ Environment, Exercise and Nutrition Study, *BMI* body mass index; ^a^ Fisher’s *Z*-Test *P* <0.001

### Quality of BMI correction models

In Table 3, the adjusted- *R*^2^ statistics were higher for models with weight as dependent variable (85.6–88.3 %) compared to models of height (67.1–77.4 %) and BMI (72.7–83.1 %), which is consistent with the pattern of observed correlations (Table 2). Across all outcomes (height, weight, BMI), the model with child and parent reports (Model 3) had the highest adjusted- *R*^2^ (higher is better) for males and females. The AIC statistic for Model 3 was consistently smaller (smaller AIC is better) compared to models with only either report, with a reduction of 10 units or more which indicates significant improvement [33]. Model equations are provided in the Appendix.

**Table 3:** Quality of correction models for height, weight, and BMI

	Males (*N* = 258)			Females (*N* = 217)		
Outcome	Parent Report	Child Report	Parent and Child Reports	Parent Report	Child Report	Parent and Child Reports
Height						
Adjusted R^2^, %	67.1	72.4	74.9	69.2	72.6	77.4
AIC	872.9	827.0	804.4	586.4	561.0	521.4
Weight						
Adjusted R^2^, %	85.6	86.4	87.6	87.3	85.5	88.3
AIC	823.8	807.9	787.0	646.1	675.3	629.2
BMI						
Adjusted R^2^, %	72.7	75.3	78.7	80.4	78.8	83.1
AIC	367.8	341.8	305.5	267.9	285.0	237.2

*BMI* body mass index, *AIC* Akaike Information Criterion

One difference between males and females is worth noting. Models with only the child report have superior fit (higher adjusted- *R*^2^, smaller AIC) compared to models with only the parent report for height, weight and BMI among males. However, models with only the parent report are superior to models with only the child report for weight and BMI among females.

### Accuracy of corrected BMI and obesity estimates

When comparing corrected estimates to raw reports, we found that cross-validated RMSEs of ‘indirect’ corrected estimates were always smaller than those of raw reports (Tables 4 and 5). As shown in Table 6, corrected means of height, weight and BMI were closer to those obtained from objective measurements, compared to child- or parent-reported means. Also, the corrected obesity and overweight/obesity prevalence estimates (Table 6) were less biased than estimates derived from raw reports (Table 1), suggesting that correction improves upon raw reports.

**Table 4:** RMSE, sensitivity, and specificity of reported and corrected outcomes computed using leave-one-out-validation

	Raw reports		Indirect Correction^d^			Direct Correction^e^		
Males (*N* = 258)	Parent Report	Child Report	Parent Report	Child Report	Both Reports^c^	Parent Report	Child Report	Both Reports^c^
Body mass index								
RMSE	2.12	2.03	1.84	1.85	1.82	2.09	1.97	1.92
Sensitivity								
Obese^a^, %	71.0	71.0	83.9	74.2	77.4	67.7	71.0	71.0
Overweight or obese^b^, %	84.6	81.5	83.1	83.1	81.5	80.0	76.9	81.5
Specificity^b^								
Obese^a^, %	98.7	99.1	98.7	98.7	99.1	99.1	99.6	99.1
Overweight or obese^b^, %	92.2	93.8	92.7	94.3	93.3	93.8	94.8	93.8

*RMSE* root mean-squared error, *BMI* body mass index; ^a^ BMI ≥95 percentile for age and gender; ^b^ BMI ≥85 percentile for age and gender; ^c^ model includes child and parent reports; ^d^ separate models for height, weight; ^e^ single model for BMI

**Table 5:** RMSE, sensitivity, and specificity of reported and corrected outcomes computed using leave-one-out-validation

	Raw reports		Indirect Correction^d^			Direct Correction^e^		
Females (*N* = 217)	Parent Report	Child Report	Parent Report	Child Report	Both Reports^c^	Parent Report	Child Report	Both Reports^c^
Body mass index								
RMSE	2.05	2.15	1.72	1.92	1.78	1.86	1.97	1.82
Sensitivity								
Obese^a^, %	85.0	70.0	90.0	85.0	85.0	85.0	65.0	85.0
Overweight or obese^b^, %	61.8	63.2	66.2	76.5	70.6	64.7	69.1	64.7
Specificity, %								
Obese^a^, %	98.5	98.5	98.5	97.0	96.5	98.5	98.0	98.0
Overweight or obese^b^, %	94.0	96.6	92.0	93.2	90.6	93.3	94.6	92.6

*RMSE* root mean-squared error, *BMI* body mass index; ^a^ BMI ≥95 percentile for age and gender; ^b^ BMI ≥85 percentile for age and gender; ^**c**^ model includes child and parent reports; ^d^ separate models for height, weight; ^e^ single model for BMI

**Table 6:** Indirect corrected estimates using dual (parent and child) reports

	Total, *N* = 475	Boys, *N* = 258	Girls, *N* = 217
Indirect Estimates (Model3^c^)			
Corrected height (cm), mean (SD)	159.3 (7.4)	160.3 (8.2)	158.1 (6.1)
Corrected weight (kg), mean (SD)	53.1 (12.0)	52.7 (12.2)	53.6 (11.7)
Corrected BMI, mean (SD)	20.8 (3.9)	20.4 (3.8)	21.4 (4.0)
Corrected obese^a^, %	10.5 ^ns^	10.1 ^ns^	11.1 ^ns^
Corrected overweight or obese^b^, %	27.0 ^ns^	25.6 ^ns^	28.6 ^ns^

*BMI* body mass index, *SD* standard deviation; ^a^ BMI ≥95 percentile for age and gender; ^b^ BMI ≥85 percentile for age and gender; ^c^ model includes child and parent reports

When comparing the ‘indirect’ and ‘direct’ correction approaches, we found that the cross-validated RMSEs from the ‘indirect’ approach were smaller than those of the ‘direct’ approach (Tables 4 and 5). While the three models for the ‘indirect’ approach had similar RMSEs for male BMI, model 3 had the smallest RMSE while ‘indirect’ model 1 had the smallest RMSE for female BMI. Generally, sensitivity of estimates from the ‘indirect approach’ was higher than those from the ‘direct approach’.

When comparing sensitivity across indirect models, results varied by gender and outcome. Indirect model 1 had the highest sensitivity for male obesity (83.9 %), and substantially higher than that of uncorrected reports (71 %). For the indicator of male overweight/obesity, uncorrected estimates from parent reports and corrected estimates from indirect models 1 and 2 had similar sensitivity (84.6 and 83.1 %, respectively). Among females, indirect model 1 (90 %) had higher sensitivity than uncorrected reports for obesity. For the indicator of overweight/obesity in females, the sensitivity of raw child and parent reports was especially low at 61.8 and 63.2 %, respectively. In comparison, indirect model 2 had the highest sensitivity (76.5 %) for female overweight/obesity.

When comparing specificity across the six models (using child report, parent report, or dual reports) under ‘indirect’ or ‘direct’ approach, we found the accuracy to be similar across all models. When comparing specificity for indicators of obesity and overweight/obesity, accuracy was slightly lower for overweight/obesity.

## Discussion

This paper aimed to assess the performance of linear correction models for BMI in child samples. Three linear models were considered – using only parent report, using only child report, using both reports. The robustness of our correction was evaluated with cross-validation. Our findings highlight that reported height and weight are unreliable, regardless of gender of child, and emphasize the importance of objective measurement. Yet objective measurements may not be feasible for many studies. When choosing between parent- and child-reports of children’s height and weight, parent-reports may be less biased, on average. However, there appears to be a non-negligible amount of bias in uncorrected parent-reports in the right tail (indicators of male obesity, female overweight/obesity), which may be of interest to researchers and practitioners. Thus, BMI correction, shown to work well in adults, may be helpful for studies of children that collect only reports.

Two important, consistent findings emerge from the cross-validated metrics of prediction performance of our correction models estimated with a sample of 12–13-year-olds. First, corrected estimates of height, weight and BMI have less error than uncorrected estimates (Table 6). Second, an ‘indirect’ approach has better performance than a ‘direct’ approach – together, these findings provide support for linear BMI correction using separate models for height and weight. The quality of our models is similar to that of correction equations published by two recent studies conducted with 17–18 year olds in France (R^2^ ranging from 67 to 87 %) [24], and with 8^th^ and 11^th^ graders enrolled in Texas schools [34].

However, the choice of which ‘indirect’ model to use – i.e. only parent-report, only child-report, or both reports is less clear. When assessing model fit, we found that there may be additional value in collecting multiple reports of height and weight. However, cross-validated RMSEs of BMI indicated that a model that includes only parent reports is most efficient for males and females, compared to a model with only child reports or a model with both reports. This difference may be explained by the fact that while AIC and leave-one-out cross validation are asymptotically equivalent, AIC can under-penalize complex models for smaller sample sizes [35]. The results for specificity, which is the proportion of those without ‘disease’ accurately classified, were generally high across all models. When assessing sensitivity for obesity and overweight/obesity, the corrected estimate did better than the raw report for three of four binary indicators. However, the best model varied by gender and outcome, and included only parent reports or only child reports. There may be considerable variation in model performance across other child characteristics (e.g. race/ethnicity, age or BMI categories) also, which were not assessed in this study.

Based on these findings, we provide guidance for future studies collecting height and weight of children. Evidence of bias in parent and child reports suggests that objective measurement is best. For researchers whose study budgets do not allow BMI measurement for all subjects, an option is to collect child reports, parent reports and objective measurements on a sub-sample, which can be used to conduct corrections for reported BMI in the sample without measurements. However, different BMI correction models may perform best for different outcomes (as suggested by our results) or subgroups of children so that a large validation sample may be necessary to identify the optimal model for each sub-group, which may be very costly. Our results in Tables 4 and 5 indicate that a model with multiple (parent and child) reports was best, or a close second, for 9 out of 10 metrics of prediction performance. Thus, a model with multiple reports may offer a multi-purpose and practical solution for BMI correction. When a validation sample cannot be obtained as part of the study design, the benefit of using correction equations developed for another dataset versus use of raw reports should be weighed.

Prior studies conducted with youth samples have typically focused on the magnitude of response biases and predictors of reporting bias rather than on BMI correction. Our descriptive analyses provided similar findings as those in the literature with some exceptions. On average, underreporting was greater for weight than height; child and parent reports of weight were less accurate for females than males [15]. Also, under-reporting of height and weight led to slight underestimation of BMI, and lower prevalence of obesity, compared to measurements. This last finding differs from Shields et al. [14] who found that estimates of obesity from parental reports and measurements were similar among 9–11 year olds. Our sample of children is slightly older, however. We also found that the sensitivity of parent-reported overweight/obese in males (84.6 %) and obese in females (85 %) in our sample was considerably higher than the sensitivity of child reported estimates (59 to 75 % for overweight/obese and 70 to 74 % for obese) from nationally representative data [15].

The few papers that have considered BMI correction in children have several limitations that we have been able to address. Two papers proposed correction models using the ‘direct’ approach [24, 36], without exploring differences in reporting errors in height and weight. Another study did not include parent-reported height and weight [34]. A well-established set of correction models for height and weight [16] extensively used in the economics of obesity literature (e.g., [37–39]) was also applied to a sample of 14–22 year olds in the National Longitudinal Survey of Youth without careful consideration of out-of-sample predictive performance. None of these applications considered the value of parent reports, although self-reported height and weight among children aged younger than 14 years have low accuracy [40–43]. Also, most have ignored the issue of validity shrinkage [23].

Our results suggest an important opportunity for improving BMI predictions when objective assessments are not available for the entire sample of children surveyed by collecting self and proxy reports of the child’s height and weight. While the sample size of our validation study did not permit stratified comparisons by child characteristics (e.g. age group, race-ethnicity or weight group), future research is needed to explore these issues.

## Limitations

Our study had several limitations. First, the sample size of the validation study was smaller than those of other correction studies, so that our statistical power was limited. It is important to replicate our results for less common outcomes (e.g. obesity) with larger sample sizes. Second, our study included 12–13 year olds with parents in the military, so that our sample may have different reporting biases than children of other ages and in the civilian population. Third, while all children who completed an M-TEENS survey were eligible for measurement, our validation sample included only those who attended on-site measurement visits. Children who were measured differed from those who were not with respect to whether they lived on base, which is to be expected given that measurements were done on-base. Reported height and weight were not significantly different for these groups, however, so that this may not be a source of systematic bias. Finally, the sensitivity of corrected estimates might be improved (100 % is the maximum) by using a measurement-error model that can account for reporting errors in surveys, or classification or regression approaches that directly model binary outcomes.

## Conclusions

Objective measurements of height and weight are always preferable over self- or proxy-reports. However, correction models generate estimates that are closer to objective measures than reports. Therefore, in situations where BMI measurement for all subjects is infeasible, an approach that combines collecting a validation sub-sample and multiple reports of adolescents’ height and weight with estimation of BMI correction models may offer a cost-effective and reasonable solution.

## Appendix

**Table 7:** Distribution of bias in child and parent reports of height, weight and BMI

	Mean (SD)	25^th^ percentile	50^th^ percentile	75^th^ percentile
Bias in Height				
Objective - parent report (cm)	−1.5 (6.2)	−2.9	−0.6	1.2
Objective - child report (cm)	−0.9 (5.8)	−2.5	−0.2	1.5
Bias in Weight				
Objective - parent report (kg)	−2.4 (4.7)	−3.6	−1.1	0.3
Objective - child report (kg)	−2.0 (4.8)	−2.9	−0.9	0.5
Bias in BMI				
Objective - parent report (kg/m^2^)	−0.5 (2.0)	−1.4	−0.3	0.4
Objective - child report (kg/m^2^)	−0.5 (2.0)	−1.2	−0.3	0.4

*BMI* body mass index, *SD* standard deviation

**Table 8:** Model equations for correction of child and parent reports of height, weight and BMI

Males (Model 1)
Height_C_ (cm) = 126.335 + 0.644 height_PR_(cm) + 0.008 height_PR_^2^(cm) + 0.206 age(months) + 2.548 race_Black_ - 0.428 race_Hispanic_ + 0.45 race_Other_
Weight_C_ (kg) = 51.582 + 0.922 weight_PR_(kg) + 0.004 weight_PR_^2^(kg) + 0.002 age(months) + 1.189 race_Black_ - 0.179 race_Hispanic_ + 0.331 race_Other_
BMI_C_ (kg/m^2^) = 21.239 + 0.812 BMI_PR_(kg/m^2^) + 0.016 BMI_PR_^2^(kg/m^2^) - 0.008 age(months) + 0.053 race_Black_ + 0.318 race_Hispanic_ + 0.217 race_Other_
Males (Model 2)
Height_C_ (cm) = 137.813 + 0.675 height_CR_(cm) + 0.009 height_CR_^2^(cm) + 0.131 age(months) + 2.697 race_Black_ - 0.076 race_Hispanic_ + 1.056 race_Other_
Weight_C_ (kg) = 55.664 + 0.917 weight_CR_(kg) + 0.002 weight_CR_^2^(kg) - 0.025 age(months) + 2.102 race_Black_ + 0.105 race_Hispanic_ + 0.844 race_Other_
BMI_C_ (kg/m^2^) = 19.854 + 0.803 BMI_CR_(kg/m^2^) + 0.019 BMI_CR_^2^(kg/m^2^) + 0.001 age(months) + 0.168 race_Black_ + 0.293 race_Hispanic_ + 0.094 race_Other_
Males (Model 3)
Height_C_ (cm) = 139.757 + 0.198 height_PR_(cm) + 0.011 height_PR_^2^(cm) + 0.516 height_CR_(cm) + 0.0 height_CR_^2^(cm) + 0.117 age(months) + 2.627 race_Black_ + 0.101 race_Hispanic_ + 0.771 race_Other_
Weight_C_ (kg) = 55.563 + 0.409 weight_PR_(kg) + 0.002 weight_PR_^2^(kg) + 0.533 weight_CR_(kg) + 0.001 weight_CR_^2^(kg) - 0.023 age(months) + 1.688 race_Black_ - 0.045 race_Hispanic_ + 0.609 race_Other_
BMI_C_ (kg/m^2^) = 21.239 + 0.393 BMI_PR_(kg/m^2^) + 0.001 BMI_PR_^2^(kg/m^2^) + 0.47 BMI_CR_(kg/m^2^) + 0.016 BMI_CR_^2^(kg/m^2^) - 0.008 age(months) + 0.069 race_Black_ + 0.254 race_Hispanic_ + 0.081 race_Other_
Females (Model 1)
Height_C_ (cm) = 141.878 + 0.644 height_PR_(cm) + 0.008 height_PR_^2^(cm) + 0.1 age(months) + 0.04 race_Black_ - 0.241 race_Hispanic_ + 0.047 race_Other_
Weight_C_ (kg) = 52.622 + 0.91 weight_PR_(kg) + 0.003 weight_PR_^2^(kg) + 0.003 age(months) + 0.212 race_Black_ - 0.313 race_Hispanic_ + 1.102 race_Other_
BMI_C_ (kg/m^2^) = 20.592 + 0.806 BMI_PR_(kg/m^2^) + 0.015 BMI_PR_^2^(kg/m^2^) + 0.003 age(months) + 0.104 race_Black_ - 0.134 race_Hispanic_ + 0.429 race_Other_
Females (Model 2)
Height_C_ (cm) = 146.923 + 0.71 height_CR_(cm) + 0.003 height_CR_^2^(cm) + 0.07 age(months) + 0.041 race_Black_ + 0.204 race_Hispanic_ - 0.342 race_Other_
Weight_C_ (kg) = 55.997 + 0.899 weight_CR_(kg) + 0.003 weight_CR_^2^(kg) - 0.017 age(months) - 0.249 race_Black_ - 0.938 race_Hispanic_ + 0.9 race_Other_
BMI_C_ (kg/m^2^) = 22.692 + 0.821 BMI_CR_(kg/m^2^) + 0.014 BMI_CR_^2^(kg/m^2^) - 0.01 age(months) -0.062 race_Black_ - 0.453 race_Hispanic_ + 0.667 race_Other_
Females (Model 3)
Height_C_ (cm) = 148.891 + 0.301 height_PR_(cm) + 0.009 height_PR_^2^(cm) + 0.45 height_CR_(cm) -0.003 height_CR_^2^(cm) + 0.055 age(months) + 0.082 race_Black_ + 0.325 race_Hispanic_ - 0.081 race_Other_
Weight_C_ (kg) = 55.634 + 0.577 weight_PR_(kg) + 0.003 weight_PR_^2^(kg) + 0.358 weight_CR_(kg) -0.001 weight_CR_^2^(kg) - 0.015 age(months) + 0.024 race_Black_ - 0.476 race_Hispanic_ + 0.846 race_Other_
BMI_C_ (kg/m^2^) = 21.838 + 0.462 BMI_PR_(kg/m^2^) + 0.011 BMI_PR_^2^(kg/m^2^) + 0.399 BMI_CR_(kg/m^2^) + 0.003 BMI_CR_^2^(kg/m^2^) - 0.005 age(months) + 0.0 race_Black_ - 0.291 race_Hispanic_ + 0.366 race_Other_

The formulae for estimating height, weight and BMI using child and parent reports, derived from Model 3, are shown below separately for boys and girls

*BMI* body mass index, *Height*_*C*_ corrected height, *Height*_*PR*_ parent reported height, *Height*_*CR*_ child reported height, *Weight*_*C*_ corrected weight, *Weight*_*PR*_ parent reported weight, *Weight*_*CR*_ child reported weight, *BMI*_*C*_ corrected BMI, *BMI*_*PR*_ parent reported BMI, *BMI*_*CR*_ child reported BMI
